# The Type and Concentration of Inoculum and Substrate as Well as the Presence of Oxygen Impact the Water Kefir Fermentation Process

**DOI:** 10.3389/fmicb.2021.628599

**Published:** 2021-02-11

**Authors:** David Laureys, Frédéric Leroy, Tom Hauffman, Marc Raes, Maarten Aerts, Peter Vandamme, Luc De Vuyst

**Affiliations:** ^1^Research Group of Industrial Microbiology and Food Biotechnology, Faculty of Sciences and Bioengineering Sciences, Vrije Universiteit Brussel, Brussels, Belgium; ^2^Research Group of Electrochemical and Surface Engineering, Faculty of Engineering Sciences, Vrije Universiteit Brussel, Brussels, Belgium; ^3^Laboratory of Microbiology, Department of Biochemistry and Microbiology, Faculty of Sciences, Ghent University, Ghent, Belgium

**Keywords:** water kefir, inoculum, substrate, oxygen, kinetics, modeling

## Abstract

Eleven series of water kefir fermentation processes differing in the presence of oxygen and the type and concentration of inoculum and substrate, were followed as a function of time to quantify the impact of these parameters on the kinetics of this process *via* a modeling approach. Increasing concentrations of the water kefir grain inoculum increased the water kefir fermentation rate, so that the metabolic activity during water kefir fermentation was mainly associated with the grains. Water kefir liquor could also be used as an alternative means of inoculation, but the resulting fermentation process progressed slower than the one inoculated with water kefir grains, and the production of water kefir grain mass was absent. Substitution of sucrose with glucose and/or fructose reduced the water kefir grain growth, whereby glucose was fermented faster than fructose. *Lacticaseibacillus paracasei* (formerly known as *Lactobacillus paracasei*), *Lentilactobacillus hilgardii* (formerly known as *Lactobacillus hilgardii*), *Liquorilactobacillus nagelii* (formerly known as *Lactobacillus nagelii*), *Saccharomyces cerevisiae*, and *Dekkera bruxellensis* were the main microorganisms present. Acetic acid bacteria were present in low abundances under anaerobic conditions and only proliferated under aerobic conditions. Visualization of the water kefir grains through scanning electron microscopy revealed that the majority of the microorganisms was attached onto their surface. Lactic acid bacteria and yeasts were predominantly associated with the grains, whereas acetic acid bacteria were predominantly associated with the liquor.

## Introduction

Water kefir is a naturally fermented beverage that is mainly produced at household level (Pothakos et al., 2016). Its fermentation process is usually started with water kefir grains (Horisberger, 1969; Laureys and De Vuyst, 2014, 2017). The water kefir grains contain around 14% (m m^–1^) dextran exopolysaccharides (EPS), are translucent, have a brittle structure, and are insoluble in water (Laureys and De Vuyst, 2017). The microorganisms responsible for the water kefir fermentation process are thought to reside mainly on the surface of the grains, encompassing bacterial and yeast cells, and their composition is influenced by the fermentation substrate and water composition (Moinas et al., 1980; Neve and Heller, 2002; Hsieh et al., 2012; Marsh et al., 2013; Laureys and De Vuyst, 2017; Laureys et al., 2018). The main water kefir microorganisms are lactic acid bacteria (LAB), yeasts, acetic acid bacteria (AAB), and bifidobacteria (Laureys and De Vuyst, 2014, 2017; Laureys et al., 2016; Eckel and Vogel, 2020; Eckel et al., 2020; Verce et al., 2020), some of which may possess probiotic properties (Romero-Luna et al., 2020). The key microorganisms have been defined as *Lentilactobacillus hilgardii* (formerly known as *Lactobacillus hilgardii*), *Liquorilactobacillus nagelii* (formerly known as *Lactobacillus nagelii*), *Lacticaseibacillus paracasei* (formerly known as *Lactobacillus paracasei*), and *Saccharomyces cerevisiae* (Laureys and De Vuyst, 2017).

Water kefir fermentation is usually carried out anaerobically but may be performed aerobically too (Laureys et al., 2018). In the case of an aerobic process, the presence of oxygen allows the proliferation of AAB after multiple backslopping steps, which results in the production of high concentrations of acetic acid. However, the short-term effects have not yet been investigated. Sucrose is usually the main substrate during water kefir fermentation and is metabolized by the microorganisms into ethanol, glycerol, lactic acid, acetic acid, mannitol, and a variety of aroma compounds (Laureys and De Vuyst, 2014, 2017). Additionally, sucrose is converted into water kefir grain dextran EPS by glucansucrases of *Lenl. hilgardii*, resulting in an increase of the water kefir grain mass during fermentation (Pidoux, 1989; Waldherr et al., 2010; Laureys and De Vuyst, 2014; Laureys et al., 2019). Water kefir grain mass can be considered as a waste stream, as the usual goal of water kefir fermentation is the production of liquor for its use as beverage. Nevertheless, the production of grains is sometimes desirable, for example to scale up a water kefir production process (Laureys et al., 2017) or for the production of novel biobased materials (Cottet et al., 2020). To reduce water kefir grain growth during fermentation, sucrose may be (partially) substituted with glucose and/or fructose, as sucrose is necessary for dextran EPS production (Monsan et al., 2001). However, the influence of these alternative substrates on the water kefir fermentation process has not been investigated yet. Additionally, decreasing the sucrose concentration could increase the water kefir grain growth as well, as glucansucrases are subjected to substrate inhibition (Hehre, 1946). Furthermore, the water kefir grain growth may also depend on the concentration of the grain inoculum, as the activity of dextran sucrase shifts from sucrose hydrolysis to dextran biosynthesis when the concentration of dextran increases (Mooser et al., 1985). Investigation of the influence of the type and concentration of the substrate and of the concentration of the grain inoculum on the water kefir grain growth will allow more control over the water kefir fermentation process.

Part of the microorganisms of the grain inoculum detaches from the water kefir grains into the liquor at the start of a fermentation process, but the majority of the microorganisms remains always associated with the grains (Laureys and De Vuyst, 2014, 2017). This suggests that the majority of the microbial metabolism during water kefir fermentation is associated with the grains, and that the concentration of the grain inoculum will determine the fermentation rate. Modeling and quantification of this effect may allow greater control over the water kefir fermentation rate in an industrial setting.

Water kefir liquor may also be used as a more convenient alternative inoculum to start the fermentation process, as it contains a substantial amount of microorganisms with a species diversity more or less similar to that on the water kefir grains (Laureys and De Vuyst, 2014, 2017). Such an innovative inoculation strategy would remove the need for water kefir grain mass altogether, and would thereby reduce waste streams. However, the metabolic and kinetic implications of this inoculation strategy have not been investigated yet.

This lack of fundamental insights into the water kefir fermentation process hampers its further industrial exploitation. Therefore, this paper aimed to quantify the impact of the presence of oxygen and the type and concentration of inoculum and substrate on the kinetics of the water kefir grain growth, substrate consumption, and metabolite production during the water kefir fermentation process. Mathematical models were fitted to the experimental data to allow the comparison of the biokinetic parameters involved.

## Materials and Methods

### Prefermentations

An inoculum of approximately 100 g of water kefir grains was obtained from the household water kefir fermentation process described before (Laureys and De Vuyst, 2014). To obtain the necessary amount of fresh water kefir grains, the inoculum was cultivated through a series of consecutive prefermentations through backslopping until >2,500 g of water kefir grain wet mass was produced. The prefermentations were performed in glass bottles (1, 2, 5, and 10 l) equipped with a polytetrafluoroethylene (PTFE) water lock. They were started by adding 10 g of sugar (Candico Bio, Merksem, Belgium), 5 g of dried figs (King Brand, Nazilli, Turkey), and 160 ml of tap water (Brussels, Belgium) per 50 g of water kefir grains. The bottles were incubated in a water bath at 21°C. Every 3 days, a backslopping practice was applied, whereby the water kefir grains were separated from the water kefir liquors by sieving and recultivated in fresh medium under the same conditions as described above.

### Fermentations

The water kefir grain mass and the water kefir liquor, obtained through the series of prefermentations mentioned above, were used to start eleven series of water kefir fermentation processes, each consisting of three bottles per sampling time point, differing in the presence of oxygen and the type and concentration of inoculum and substrate, encoded as explained in Table 1. The fermentations were performed in 100-ml glass bottles. Each fermentation bottle contained 85 ml of autoclaved (121°C, 2.1 bar, 20 min) water kefir simulation medium (WKSM). The WKSM was composed of 65 ml of tap water (Brussels, Belgium) and 20 ml of fig extract, supplemented with 3 (fermentation series 1S-2G-An), 6 (2S-2L-An, 2S-2G-An, 2S-2G-Ae, and 2S-3G-An), or 9 g (3S-2G-An) of sucrose (Merck, Darmstadt, Germany); 6 g of glucose (Merck; 2G-2G-An); 6 g of fructose (Merck; 2F-2G-An); 3 g of sucrose, 1.5 g of glucose, and 1.5 g of fructose (2SGF-2G-An); or 3 g of glucose and 3 g of fructose (2GF-2G-An). The concomitant concentrations of the carbohydrates added for the different fermentation series are represented in Table 1. The fig extract was prepared as described before (Laureys and De Vuyst, 2014). To start the fermentation processes, 15.0 ml of liquor inoculum (2S-2L-An); or 7.5 (2S-1G-An), 15 (2S-2G-An, 2S-2G-Ae, 1S-2G-An, 3S-2G-An, 2SGF-2G-An, 2GF-2G-An, 2G-2G-An, and 2F-2G-An), or 22.5 g (2S-3G-An) of grain inoculum was added to the fermentation bottles. Fermentation bottles were equipped with a PTFE water lock for fermentation under anaerobic conditions (2S-2L-An, 2S-2G-An, 1S-2G-An, 3S-2G-An, 2S-1G-An, 2S-3G-An, 2SGF-2G-An, 2GF-2G-An, 2G-2G-An, and 2F-2G-An) or were covered with a sterile muslin cloth for fermentation under aerobic conditions (2S-2G-Ae). All fermentation bottles were incubated in an air-conditioned room at 21°C. The contents of the fermentation bottles were mixed by gently turning the bottles at the start of the fermentation processes and before their sampling.

**TABLE 1:** Composition of the water kefir simulation media and atmospheric conditions used for eleven series of water kefir fermentation processes.

Fermentation series (carbohydrates-inoculum-oxygen conditions)	Sucrose (S, g l^–1^)	Glucose (G, g l^–1^)	Fructose (F, g l^–1^)	Inoculum (G, grains; L, liquor)	Oxygen conditions (An, anaerobic; Ae, aerobic)
2S-2G-An	71	0	0	15 g of G	An
2S-2L-An	71	0	0	15 ml of L	An
2S-2G-Ae	71	0	0	15 g of G	Ae
2S-1G-An	71	0	0	7.5 g of G	An
2S-3G-An	71	0	0	22.5 g of G	An
1S-2G-An	35	0	0	15 g of G	An
3S-2G-An	106	0	0	15 g of G	An
2SGF-2G-An	35	18	18	15 g of G	An
2GF-2G-An	0	35	35	15 g of G	An
2G-2G-An	0	71	0	15 g of G	An
2F-2G-An	0	0	71	15 g of G	An

*The concentrations of the carbohydrates added exclude those of the fig extract.*

### Visualization of the Water Kefir Grains

To study the microbial colonization of the water kefir grains, grain samples of the household water kefir fermentation process mentioned above were brought into tubes, rinsed twice by adding 1 ml of 0.05 M phosphate buffer (PB) at pH 7.2, incubated for 10 min at room temperature, and the supernatant was removed. The samples were fixated with 1 ml of 2.5% (m v^–1^) glutaraldehyde solution in PB and incubated for 10 min at room temperature, after which the supernatants were removed. This fixating procedure was repeated with an incubation time of 18 h. Afterward, the samples were rinsed twice with PB as described above. The samples were dehydrated by consecutively adding 1 ml of 50, 70, 90, and twice 100% (v v^–1^) of ethanol (diluted in ultrapure water), incubating for 20 min at room temperature, and removing the supernatant. The samples were dried by adding 500 μl of hexamethyldisilazane (Sigma-Aldrich, St. Louis, MO, United States), incubated for 1 h at room temperature, and the supernatant was removed. This drying procedure was repeated, after which the water kefir grain samples were dried for 12 h at room temperature under vacuum.

The water kefir grain samples were fixed on the sample holder with carbon tape and coated with 3.0 nm of gold with a Cressington 208HR sputter coater (Cressington Scientific Instruments, Watford, United Kingdom). Afterward, the sample was loaded under high vacuum in a JSM-IT300 scanning electron microscope for visualization (Jeol Europe, Nieuw-Vennep, Netherlands).

### Analyses

After 0, 1, 2, 3, and 4 days of fermentation for all fermentation series, as well as after 6 days of fermentation for fermentation series 2S-2L-An, 2S-2G-Ae, 2S-1G-An, and 3S-2G-An, three fermentation bottles (representing three independent biological replicates) were removed and their contents were analyzed. The pH, the water kefir grain wet mass, and the concentrations of the substrates and metabolites were determined at every sampling time. The viable counts of the LAB, yeasts, and AAB were determined in the water kefir liquor and grain inocula, and in the liquors and grains of fermentation series 2S-2G-An, 2S-2L-An, and 2S-2G-Ae after 4 days of fermentation. The culture-dependent microbial species diversities of the LAB, yeasts, and AAB were determined in the water kefir liquor and grain inocula. Those of the AAB were also determined in the water kefir liquors of fermentation series 2S-2G-An, 2S-2L-An, and 2S-2G-Ae after 4 days of fermentation. The culture-independent microbial species diversities were determined in the water kefir liquor and grain inocula, and in the water kefir liquors and grains of fermentation series 2S-2G-An, 2S-2L-An, 2S-2G-Ae, 2GF-2G-An, 2G-2G-An, and 2F-2G-An after 4 days of fermentation. The results are presented as the mean ± standard deviation of the three independent biological replicates performed for each fermentation series at each sampling point, if applicable.

### pH, Water Kefir Grain Wet Mass, and Water Kefir Grain Density Determinations

The pH, the water kefir grain wet mass, and the water kefir grain growth were determined as described previously (Laureys et al., 2018). The density of the water kefir grains was determined in triplicate with a volumetric flask of 1.00 l. Hereto, its exact volume was determined by weighing the volumetric flask when empty and when filled with ultrapure water at 21°C. Approximately 280 g of water kefir grains were brought into the empty flask, which was then filled with ultrapure water at 21°C. The water kefir grain density was calculated based on the volume of the flask, the mass of the water kefir grains, and the mass of ultrapure water needed to fill the flask containing water kefir grain mass.

### Microbial Enumerations

The viable counts of the presumptive LAB were determined on de Man-Rogosa-Sharpe (MRS) agar medium, those of the presumptive AAB on modified deoxycholate-mannitol-sorbitol (mDMS) agar medium, and those of presumptive yeasts on yeast extract-peptone-dextrose (YPD) agar medium, as described before (Laureys et al., 2018).

### Culture-Dependent Microbial Species Diversity and Community Dynamics Analyses

The culture-dependent microbial species diversities of the LAB, yeasts, and AAB in the water kefir liquors and grains were determined by randomly picking 10 to 20% of the total number of colonies from the respective agar media with 30 to 300 colonies. The isolates were subcultivated on their respective agar media until the third generation, which was used for dereplication *via* matrix-assisted laser desorption/ionization time-of-flight mass spectrometry (MALDI-TOF MS) fingerprinting, as described before (Laureys et al., 2018). The peptide fingerprint patterns obtained were clustered numerically by means of the BioNumerics software version 7.50 (Applied Maths, Sint-Martens-Latem, Belgium). Representative bacterial isolates within each cluster were identified by sequencing part of their 16S rRNA gene from genomic DNA, and representative yeast isolates within each cluster were identified by sequencing part of their 26S large subunit (LSU) rRNA gene and internal transcribed spacer (ITS) region from genomic DNA, as described previously (Laureys et al., 2018).

### Exopolysaccharide Production

All bacterial isolates were grown on MRS agar medium supplemented with 10 g l^–1^ of sucrose at 30°C for 7 days to visually assess their EPS production capacity.

### Culture-Independent Microbial Species Diversity and Community Dynamics Analyses

The culture-independent microbial species diversities of bacteria and yeasts in the water kefir liquors and grains were determined after preparing total DNA extracts from the cell pellets of the water kefir liquors and 0.2 g of crushed water kefir grains, respectively, as described previously (Laureys et al., 2018). The culture-independent microbial community profiles were obtained by amplifying selected genomic fragments in the total DNA with the universal prokaryotic primer pair (V3), the LAB-specific primer pair (LAC), the *Bifidobacterium*-specific primer pair (Bif), and the universal eukaryotic primer pair (Yeast); and separating the PCR amplicons through denaturing gradient gel electrophoresis (DGGE), as described previously (Laureys and De Vuyst, 2014). Selected bands of the community profiles were cut from the gels and identities were assigned through sequencing, as described previously (Laureys and De Vuyst, 2014).

### Substrate and Metabolite Concentration Determinations

Samples for substrate and metabolite concentration analyses were prepared as described previously (Laureys and De Vuyst, 2014). The concentrations of sucrose, glucose, fructose, glycerol, and mannitol were determined through high-performance anion exchange chromatography with pulsed amperometric detection (HPAEC-PAD), those of D- and L-lactic acid and acetic acid through high-performance liquid chromatography with ultraviolet detection (HPLC-UV), those of ethanol through gas chromatography with flame ionization detection (GC-FID), and those of the aroma compounds through static headspace gas chromatography with mass spectrometry detection (SH-GC-MS), as described previously (Laureys and De Vuyst, 2014).

### Statistics

An ANOVA was performed to test for differences between the eleven fermentation series, followed by a series of *post hoc* pairwise comparisons with Fisher’s least significant difference (LSD) test, as described previously (Laureys et al., 2019). All statistical tests were performed in R 3.2.0 with a significance level of 0.05.

## Kinetic Model Development

### Model Equations

To compare the kinetics of different water kefir fermentation processes, a mathematical model was developed as follows. During water kefir fermentation, sucrose could be converted into glucose and fructose by invertases, or could be converted by glucansucrases into fructose and suspended EPS (EPS_Liquor_) or grain EPS (EPS_Grains_). The production of water kefir grain wet mass as a function of time during water kefir fermentation could be described by a logistic model with a maximum specific water kefir grain production rate k_EPS_Grains_ (h^–1^; g of grain wet mass per liter per hour per g of grain wet mass per liter) and a maximal water kefir grain wet mass concentration [EPS_Grains_max_] (g l^–1^), in analogy with a report on milk kefir grain growth (Zajšek and Goršek, 2010):

(1) $$\frac{d[EPS_{Grains}]}{dt}=k_{EPS\_Grains}*\left(1-\frac{\left[EPS_{Grains}\right]}{\left[EPS_{Grains\_max}\right]}\right)*[EPS_{Grains}]$$

This differential equation was solved with [EPS_Grains_] = [EPS_Grains___0_] when *t* = 0 h, resulting in a non-linear model.

The concentrations of ethanol (Eth), glycerol (Gly), lactic acid (LA), acetic acid (AA), and mannitol (Mtl) (g l^–1^) were described as a function of time with their initial concentrations [Eth_0_], [Gly_0_], [LA_0_], [AA_0_], and [Mtl_0_] (g l^–1^), and their volumetric production rates k_Eth_, k_Gly_, k_LA_, k_AA_, and k_Mtl_ (g l^–1^ h^–1^). This could be illustrated *via* a general expression for each metabolite (P), as follows:

(2) $$\left[P\right]=\left[P_{0}\right]+k_{P}*t$$

To estimate the initial concentrations and volumetric production rates for all fermentation series, a linear model was developed, whereby the initial concentrations depended on the concentration inoculum (Inoculum) and the volumetric production rates depended on the fermentation series (Time:Series):

(3) P∼I⁢n⁢o⁢c⁢u⁢l⁢u⁢m+T⁢i⁢m⁢e:S⁢e⁢r⁢i⁢e⁢s

The consumption of glucose and fructose as a function of time was only described for the fermentation series without sucrose. Experimental data from fermentation series containing sucrose were not modeled, due to the complexity related to the release of either fructose (glucansucrase) or glucose and fructose (invertase) from sucrose. The consumption of glucose and/or fructose for the production of each metabolite was described by a conversion factor, which represented the theoretical mass of glucose (or fructose) consumed for the production of a certain mass of metabolite (g g^–1^). The production of ethanol and acetic acid due to yeast and LAB metabolism, respectively, were assumed to release equimolar amounts of carbon dioxide. The consumption of glucose and/or fructose for the production of metabolites and compounds that were not measured, such as biomass, was described with a volumetric production rate k_Rest_ (g l^–1^ h^–1^). When the initial concentrations of glucose and fructose were similar, glucose was consumed faster than fructose (see below). To describe the faster consumption of glucose (Glc) compared to fructose (Fru), a dimensionless glucose preference factor (P_Glc_) was introduced.

(4) $$\frac{d\,\left[G\,l\,c\right]}{d\,t}=-\frac{\overset{(1.96k_{E\,t\,h}+0.98k_{G\,l\,y}+1.00k_{L\,A}+1.50k_{A\,A}}{+0.99k_{M\,t\,l}+k_{R\,e\,s\,t})*P{}_{G\,l\,c}*[Glc]}}{P_{G\,l\,c}*\left[G\,l\,c\right]+\left[F\,r\,u\right]}$$

(5) $$\frac{d\,\left[F\,r\,u\right]}{d\,t}=-\frac{\overset{(1.96k_{E\,t\,h}+0.98k_{G\,l\,y}+1.00k_{L\,A}+1.50k_{A\,A}}{+0.99k_{M\,t\,l}+k_{R\,e\,s\,t})*[Fru]}}{P_{G\,l\,c}*\left[G\,l\,c\right]+\left[F\,r\,u\right]}$$

### Fitting of the Models to the Experimental Data

The parameters for the production kinetics of the water kefir grain mass and the metabolites were estimated by fitting the above-described non-linear and linear models, respectively, to the experimental data. The volumetric production rates (k_P_) for the production of measured metabolites and non-measured compounds, and the glucose preference factor (P_Glc_), were estimated by solving the above-mentioned set of differential equations. All calculations were performed in R 3.2.0. The estimations of the biokinetic parameters are presented as the mean ± standard error.

The model parameters of Eq. 1, describing the production of EPS during the water kefir fermentation processes, were estimated by fitting a non-linear model to the experimental data obtained after 0, 24, 48, 72, and 96 h of fermentation for all fermentation series containing sucrose. The values of [P_0_] and k_P_ were estimated for each metabolite by fitting a linear model to the linear portions of the experimental data, which was from 0 to 72 h (see below), for all fermentation series. The values of k_Rest_ were estimated by fitting the set of differential equations to the experimental data of fermentation series 2GF-2G-An, 2G-2G-An, and 2F-2G-An after 0, 24, 48, and 72 h of fermentation. The value of P_Glc_ was estimated by fitting the set of differential equations to the experimental data of the fermentation series 2GF-2G-An obtained after 0, 24, 48, and 72 h of fermentation.

## Results

### Water Kefir Grain Density and Visualization of the Water Kefir Grains

**FIGURE 1:**
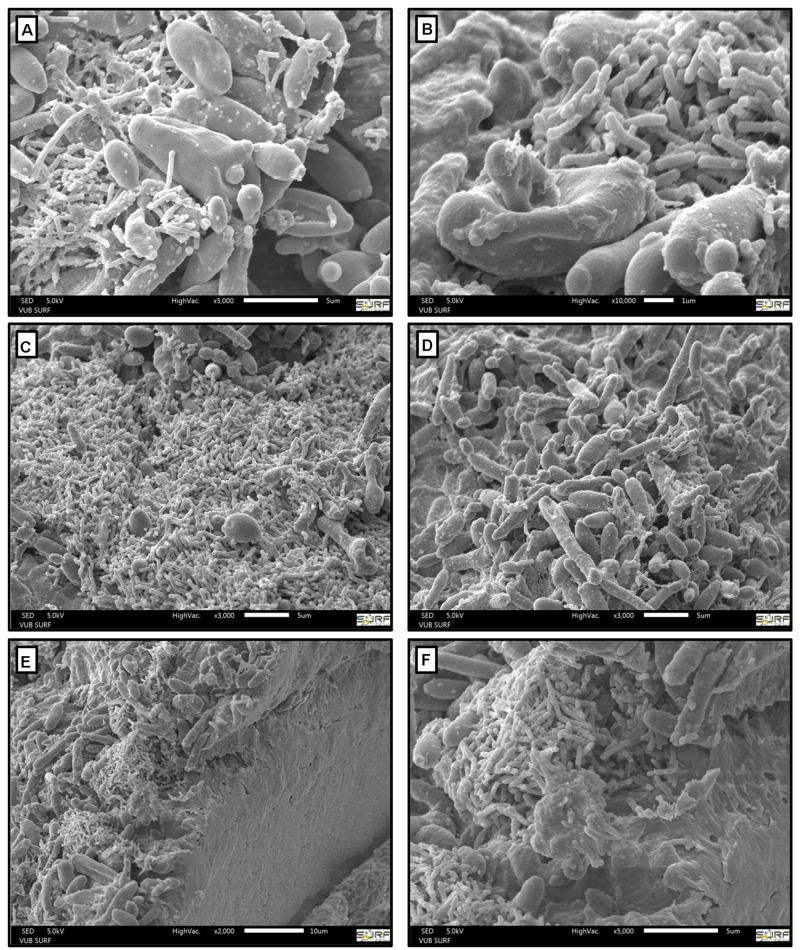
Scanning electron microscopy images of water kefir grains. Visualization of two different locations on the water kefir grain surfaces with a magnification level of 5,000 **(A)**, 10,000 **(B)**, and 3,000 **(C,D)**, and visualization of the inside of a water kefir grain with a magnification level of 2,000 **(E)** and 5,000 **(F)**. The sizes are indicated with horizontal white bars.

The density of the water kefir grains was 1.0495 ± 0.0004 g ml^–1^. Visualization of the water kefir grains *via* scanning electron microscopy revealed that their surface was covered with microorganisms (Figures 1A,B). Yeasts and LAB were found as mixed consortia, whereby some areas were predominantly occupied by either LAB (Figure 1C) or yeasts (Figure 1D). When a water kefir grain was cut with a sterile scalpel, no discernible microorganisms could be found inside the grains (Figures 1E,F).

### Microbial Enumerations

The viable counts of the yeasts on the grain inoculum were similar to those on the grains of the anaerobic (2S-2G-An) and aerobic fermentation series (2S-2G-Ae) after 4 days of fermentation (Table 2). The viable counts of the yeasts in the liquor inoculum were similar to those in the liquors of the anaerobic (2S-2G-An) and aerobic (2S-2G-Ae) fermentation series, and to those in the liquors of the fermentation series performed with a liquor inoculum (2S-2L-An) after 4 days of fermentation. Likewise, the viable counts of the LAB on the grain inoculum were similar to those on the grains of the anaerobic (2S-2G-An) and aerobic (2S-2G-Ae) fermentation series. The viable counts of the LAB in the liquor inoculum and in the liquors of the fermentation series performed with a liquor inoculum (2S-2L-An) were higher than those in the liquors of the anaerobic (2S-2G-An) and aerobic (2S-2G-Ae) fermentation series. The viable counts of the AAB were higher on the grains of the aerobic fermentation series (2S-2G-Ae) than on those of the grain inoculum and the anaerobic fermentation series (2S-2G-An). The viable counts of the AAB were highest in the liquors of the aerobic fermentation series (2S-2G-Ae), lower in the liquors of the fermentation series inoculated with a liquor inoculum (2S-2L-An), and lowest in the liquor inoculum and in the liquors of the anaerobic fermentation series (2S-2G-An).

**TABLE 2:** Viable counts of the yeasts, lactic acid bacteria (LAB), and acetic acid bacteria (AAB) in the liquor (log cfu ml^–1^) and grain inocula (log cfu g^–1^), and in the liquors (log cfu ml^–1^) and on the grains (log cfu g^–1^) of fermentation series 2S-2G-An, 2S-2L-An, and 2S-2G-Ae after 4 days of fermentation, as well as the ratios between these values.

Viable counts or ratio		Inoculum	2S-2G-An	2S-2L-An	2S-2G-Ae
Yeasts	Liquor	7.0 ± 0.1^a^	6.8 ± 0.1^b^	7.0 ± 0.1^a^	6.7 ± 0.1^b^
	Grains	7.5 ± 0.1^a^	7.3 ± 0.1^b^	NA	7.5 ± 0.1^a^
LAB	Liquor	8.0 ± 0.1^a^	7.2 ± 0.1^c^	7.8 ± 0.1^b^	7.1 ± 0.1^c^
	Grains	8.3 ± 0.1^a^	8.0 ± 0.1^b^	NA	7.9 ± 0.1^b^
AAB	Liquor	3.4 ± 0.1^c^	3.2 ± 0.3^c^	4.09 ± 0.1^b^	5.8 ± 0.1^a^
	Grains	2.9 ± 0.2^b^	2.5 ± 0.3^c^	NA	5.3 ± 0.1^a^
LAB/yeasts	Liquor	10.0 ± 2.2^a^	2.3 ± 0.3^c^	6.1 ± 0.5^b^	2.3 ± 0.2^c^
	Grains	6.3 ± 1.4^a^	4.2 ± 0.7^b^	NA	2.7 ± 0.1^c^
Grains/liquor	Yeasts	3.0 ± 0.5^b^	3.6 ± 0.9^b^	NA	6.2 ± 1.6^a^
	LAB	1.9 ± 0.6^b^	6.5 ± 0.7^a^	NA	7.3 ± 1.3^a^
	AAB	0.32 ± 0.13^a^	0.17 ± 0.03^b^	NA	0.31 ± 0.04^a^

*The results are presented as the mean ± standard deviation; significant differences (*p* < 0.05) between the series are indicated with superscripts a, b, and c. Abbreviations are as in Table 1. NA, not available.*

The ratios of the viable counts of the yeasts on the water kefir grains to those in the liquors were always around 3 and this was also the case for the LAB. In contrast, the ratios of those of the AAB on the water kefir grains to those in the liquors were always below 1. The ratios of those of the LAB to those of the yeasts were always between 2 and 10.

### Culture-Dependent Microbial Species Diversity and Community Dynamics

**FIGURE 2:**
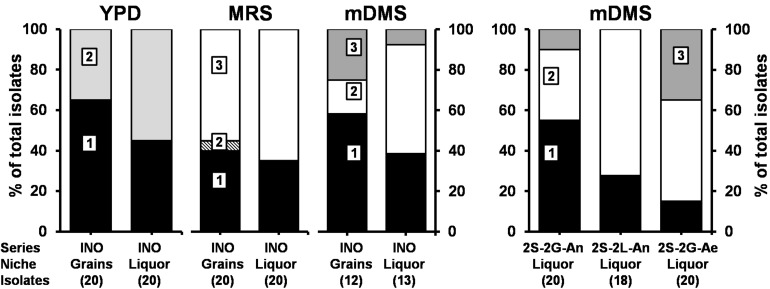
Culture-dependent species diversity for the water kefir grain and liquor inocula (INO), and for the water kefir liquors of fermentation series 2S-2G-An, 2S-2L-An, and 2S-2G-Ae after 4 days of fermentation. The number of isolates are indicated between brackets. Isolates from YPD agar medium: (1) *Saccharomyces cerevisiae* [LSU (99% identity; GenBank accession no. CP011558) and ITS (99% identity; accession no. KC515374)]; and (2) *Dekkera bruxellensis* [LSU (99% identity; accession no. GU291284) and ITS (99% identity; accession no. FJ545249)]. Isolates from MRS agar media: (1) *Lacticaseibacillus paracasei* (100% identity with *Lactobacillus paracasei*; accession no. AP012541); (2) *Lentilactobacillus hilgardii* (100% identity with *Lactobacillus hilgardii*; accession no. LC064898); and (3) *Liquorilactobacillus nagelii* (99% identity with *Lactobacillus nagelii*; accession no. NR112754). Isolates from mDMS agar media: (1) *Gluconobacter roseus*/*oxydans* (100% identity for both species; accession no. NR041049/NR026118); (2) *Acetobacter fabarum* (100% identity; accession no. NR113556); and (3) *Acetobacter indonesiensis* (99% identity; accession no. NR113847). LSU, large subunit rRNA gene; ITS, internal transcribed spacer. Abbreviations are as in Table 1.

*Saccharomyces cerevisiae* and *Dekkera bruxellensis* were the only yeast species found culture-dependently in the grain and liquor inocula, whereby the relative abundance of *D*. *bruxellensis* was higher in the liquor than on the grains (Figure 2). *Lacticaseibacillus paracasei* and *Liql*. *nagelii* were the main LAB species found culture-dependently in the liquor and grain inocula, whereas *Lenl. hilgardii* (of which 50% of the isolates produced EPS) was only found in the grain inoculum.

*Gluconobacter roseus*/*oxydans*, *Acetobacter fabarum*, and *Acetobacter indonesiensis* were found culture-dependently in the grain and liquor inocula, whereby the relative abundances of *A*. *fabarum* were higher in the liquors and those of *A*. *indonesiensis* were higher on the grains. After 4 days of fermentation, *G. roseus*/*oxydans* and *A. fabarum* were found in the liquors of fermentation series 2S-2G-An, 2S-2L-An, and 2S-2G-Ae; and *A. indonesiensis* was found in the liquors of fermentation series 2S-2G-An and 2S-2G-Ae. The relative abundances of *A*. *fabarum* were higher in the fermentation series 2S-2L-An than in 2S-2G-An and 2S-2G-Ae, and those of *G. roseus*/*oxydans* were higher in fermentation series 2S-2G-An and 2S-2L-An than in 2S-2G-Ae.

### Culture-Independent Microbial Species Diversity and Community Dynamics

**FIGURE 3:**
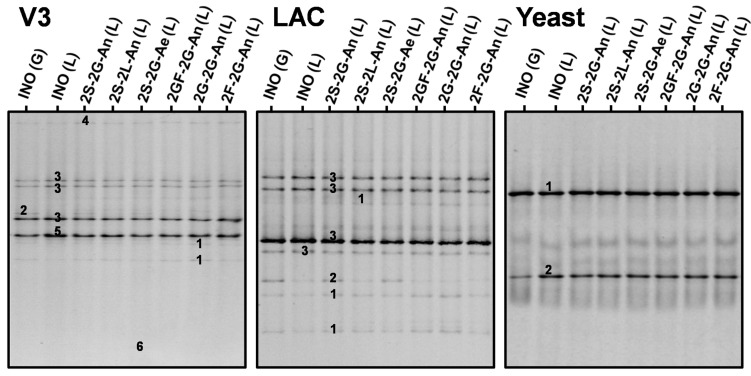
Culture-independent species diversity for the grain (G) and liquor (L) inocula (INO), and for the water kefir liquors (L) of fermentation series 2S-2G-An, 2S-2L-An, 2S-2G-Ae, 2GF-2G-An, 2G-2G-An, and 2F-2G-An after 4 days of fermentation. The numbers indicate the bands that were sequenced and the closest known type strains of the sequenced fragments are given. With the V3 primer pair: (1) *Lacticaseibacillus casei/paracasei/rhamnosus* (99% identity with *Lactobacillus casei*/*paracasei*/*zeae*/*rhamnosus* for all species; GenBank accession nos. LC064894/AB289229/AB289313/JQ580982); (2) *Lentilactobacillus hilgardii/diolivorans* (100% identity with *Lactobacillus hilgardii*/*diolivorans*; accession nos. LC064898/NR037004); (3) *Liquorilactobacillus nagelii/ghanensis* (99% identity with *Lactobacillus nagelii*/*ghanensis*; accession nos. NR119275/NR043896); (4) *Oenococcus sicerae* (99% identity; accession no. CP029684); (5) *Bifidobacterium aquikefiri* (100% identity; accession no. LN849254); (6) *Acetobacteraceae* sp. (100% identity). With the LAC primer pair: (1) *Lacc. casei*/*paracasei* (99% identity with *Lb. casei/paracasei/zeae*; accession nos. LC064894/AB289229/AB289313); (2) *Lenl. hilgardii* (100% identity with *Lb. hilgardii*; accession no. LC064898); and (3) *Liql. nagelii* (99% identity with *Lb. nagelii*; accession no. NR119275). With the Yeast primer pair: (1) *Saccharomyces cerevisiae* (100% identity; accession no. NG042623); and (2) *Dekkera bruxellensis* (100% identity; accession no. AY969049). Abbreviations are as in Table 1.

The main bands in the rRNA-PCR-DGGE community profiles obtained with the Yeast primer pair for the liquor and grain inocula were attributed to *S*. *cerevisiae* and *D*. *bruxellensis*, whereby the relative intensities of the bands attributed to *D*. *bruxellensis* were higher for the liquor inoculum than for the grain inoculum (Figure 3). Furthermore, the community profiles obtained with the Yeast primer pair for the water kefir liquors of fermentation series 2S-2G-An, 2S-2L-An, 2S-2G-Ae, 2GF-2G-An, 2G-2G-An, and 2F-2G-An were similar to those for the liquor inoculum.

The main bands in the community profiles obtained with the V3 and LAC primer pairs for the liquor and grain inocula were attributed to *Lacc*. *paracasei*, *Liql*. *nagelii*, and *Lenl*. *hilgardii*, whereby the relative intensities of the bands attributed to *Lenl*. *hilgardii* were higher for the grain inoculum than for the liquor inoculum (Figure 3). Furthermore, the relative intensities of the bands attributed to *Lenl. hilgardii* were higher for the liquors of fermentation series 2S-2G-An and 2S-2G-Ae than for those of fermentation series 2S-2L-An, 2GF-2G-An, 2G-2G-An, and 2F-2G-An. Bands with low relative intensities attributed to *Oenococcus sicerae* and bands with high relative intensities attributed to *Bifidobacterium aquikefiri* were present in the community profiles obtained with the V3 primer pair for the liquor and grain inocula, and for the liquors of fermentation series 2S-2G-An, 2S-2L-An, 2S-2G-Ae, 2GF-2G-An, 2G-2G-An, and 2F-2G-An. A band with low relative intensity attributed to the taxon *Acetobacteraceae* was found in the community profiles obtained with the V3 primer pair for the liquors of the fermentation series 2S-2G-Ae, but not for the liquor inoculum and the liquors of the other fermentation series.

### Substrate Consumption and Metabolite Production Profiles

The concentrations of the water kefir grain wet mass (Supplementary Figure S1), the substrates (Supplementary Figure S2), and the metabolites (Supplementary Figure S3) as a function of time during the eleven series of water kefir fermentation processes were fitted by the kinetic models described above. The pH always followed a similar pattern and was mainly influenced by the type and concentration of the inoculum.

**FIGURE 4:**
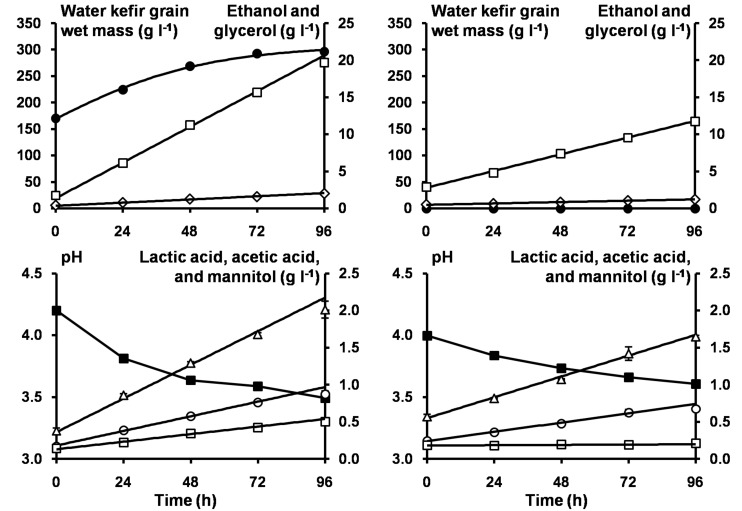
The pH (■) and concentrations of water kefir grain wet mass (∙), ethanol (□), glycerol (◆), lactic acid (Δ), acetic acid (∘), and mannitol (□) as a function of time for the anaerobic water kefir fermentation series 2S-2G-An with sucrose as substrate and started with a grain inoculum (left), and for the anaerobic water kefir fermentation series 2S-2L-An with sucrose as substrate and started with a liquor inoculum (right). The model lines (solid lines) describe the modeled concentrations of water kefir grain wet mass, ethanol, glycerol, lactic acid, acetic acid, and mannitol during the first 72 h of fermentation. Abbreviations are as in Table 1.

The model describing the production of EPS during water kefir fermentation is illustrated for the fermentation series 2S-2G-An (Figure 4). When the concentrations of the grain inoculum increased, k_EPS_Grains_ and [EPS_Grains_max_] increased (Table 3), whereas the water kefir grain growth (%) at the end of fermentation decreased (Supplementary Figure S1). When the concentrations of sucrose decreased or when sucrose was partially substituted with glucose and fructose, k_EPS_Grains_ increased and [EPS_Grains_max_] decreased, and the water kefir grain growth (%) at the end of fermentation decreased. When the fermentation processes were performed aerobically, the water kefir grain growth was similar to that of the fermentation processes under anaerobic conditions. When sucrose was substituted completely, the water kefir grain growth was zero.

**TABLE 3:** Estimated values of the model parameters during eleven series of water kefir fermentation processes differing in the presence of oxygen and the type and concentration of the inoculum and substrate: initial concentrations of water kefir grain wet mass ([EPS_Grains___0_]), maximum concentrations of water kefir grain wet mass ([EPS_Grains_max_]), and maximum specific water kefir grain production rates (k_EPS_Grains_) for the logistic models describing the concentrations of the water kefir grain wet mass as a function of time; initial concentrations and volumetric production rates of ethanol ([Eth_0_] and k_Eth_), glycerol ([Gly_0_] and k_Gly_), lactic acid ([LA_0_] and k_LA_), acetic acid ([AA_0_] and k_AA_), and mannitol ([Mtl_0_] and k_Mtl_) for the linear models describing their concentrations as a function of time; volumetric production rates of the metabolites and compounds that were not measured (k_Rest_) for the models describing their production as a function of time; estimated values for the glucose preference factor (P_Glc_) describing the consumption of glucose and fructose as a function of time; and the ratios of the volumetric production rates for the production of glycerol to ethanol, lactic acid to ethanol, acetic acid to ethanol, and acetic acid to lactic acid.

Model parameter	2S-2G-An	2S-2L-An	2S-2G-Ae	2S-3G-An	2S-1G-An	1S-2G-An	3S-2G-An	2SGF-2G-An	2GF-2G-An	2G-2G-An	2F-2G-An
(g l^–1^)	169 ± 1 ^b^	NA	169 ± 1^b^	231 ± 2^a^	93 ± 1^c^	169 ± 1^b^	169 ± 1^b^	169 ± 1^b^	NA	NA	NA
[EPS_Grains_max_] (g l^–1^)	308 ± 3^c^	NA	318 ± 4^b^	354 ± 2^a^	247 ± 3^d^	245 ± 2^d^	352 ± 4^a^	240 ± 2^d^	NA	NA	NA
k_EPS_Grains_ (x 10^–3^ h^–1^)	35 ± 2^cd^	NA	34 ± 2^d^	46 ± 2^ab^	32 ± 1^d^	50 ± 4^a^	30 ± 1^d^	41 ± 4^bc^	NA	NA	NA
[Eth_0_] (g l^–1^)	1.35 ± 0.05^c^	2.78 ± 0.16^a^	1.35 ± 0.05^c^	2.26 ± 0.16^b^	0.44 ± 0.16^d^	1.35 ± 0.05^c^	1.35 ± 0.05^c^	1.35 ± 0.05^c^	1.35 ± 0.05^c^	1.35 ± 0.05^c^	1.35 ± 0.05^c^
[Gly_0_] (g l^–1^)	0.33 ± 0.01^b^	0.47 ± 0.02^a^	0.33 ± 0.01^b^	0.44 ± 0.02^a^	0.22 ± 0.02^c^	0.33 ± 0.01^b^	0.33 ± 0.01^b^	0.33 ± 0.01^b^	0.33 ± 0.01^b^	0.33 ± 0.01^b^	0.33 ± 0.01^b^
[LA_0_] (g l^–1^)	0.36 ± 0.01^b^	0.55 ± 0.02^a^	0.36 ± 0.01^b^	0.55 ± 0.02^a^	0.19 ± 0.02^c^	0.36 ± 0.01^b^	0.36 ± 0.01^b^	0.36 ± 0.01^b^	0.36 ± 0.01^b^	0.36 ± 0.01^b^	0.36 ± 0.01^b^
[AA_0_] (g l^–1^)	0.18 ± 0.01^b^	0.24 ± 0.01^a^	0.18 ± 0.01^b^	0.24 ± 0.01^a^	0.09 ± 0.01^c^	0.18 ± 0.01^b^	0.18 ± 0.01^b^	0.18 ± 0.01^b^	0.18 ± 0.01^b^	0.18 ± 0.01^b^	0.18 ± 0.01^b^
[Mtl_0_] (g l^–1^)	0.13 ± 0.01^b^	0.18 ± 0.01^a^	0.13 ± 0.01^b^	0.16 ± 0.01^a^	0.09 ± 0.01^c^	0.13 ± 0.01^b^	0.13 ± 0.01^b^	0.13 ± 0.01^b^	0.13 ± 0.01^b^	0.13 ± 0.01^b^	0.13 ± 0.01^b^
k_Eth_ (mg l^–1^ h^–1^)	201 ± 2^bc^	94 ± 3^i^	207 ± 2^b^	231 ± 3^a^	162 ± 3^h^	189 ± 3^e^	198 ± 2^cd^	192 ± 2^de^	180 ± 2^f^	178 ± 2^fg^	172 ± 2^g^
k_Gly_ (mg l**^–^**^1^ h^–1^)	17.9 ± 0.1^bc^	7.6 ± 0.4^g^	18.7 ± 0.2^b^	20.0 ± 0.4^a^	14.9 ± 0.4^f^	16.7 ± 0.2^de^	17.4 ± 0.2^cd^	17.2 ± 0.2^cd^	16.0 ± 0.2^e^	16.7 ± 0.2^de^	14.6 ± 0.2^f^
k_LA_ (mg l^–1^ h^–1^)	18.9 ± 0.3^b^	11.7 ± 0.5^g^	18.0 ± 0.3^bcd^	20.3 ± 0.5^a^	15.0 ± 0.5^f^	18.5 ± 0.3^bc^	18.3 ± 0.3^bc^	17.1 ± 0.3^de^	16.1 ± 0.3^e^	17.6 ± 0.3^cd^	14.8 ± 0.3^f^
k_AA_ (mg l^–1^ h^–1^)	8.2 ± 0.2^c^	5.2 ± 0.3^g^	9.0 ± 0.2^b^	9.7 ± 0.3^a^	6.6 ± 0.3^f^	8.2 ± 0.2^c^	8.0 ± 0.2^cd^	7.4 ± 0.2^de^	7.2 ± 0.2^e^	7.4 ± 0.2^e^	7.1 ± 0.2^ef^
k_Mtl_ (mg l^–1^ h^–1^)	4.25 ± 0.09^b^	0.17 ± 0.13^f^	3.84 ± 0.09^c^	4.91 ± 0.13^a^	2.72 ± 0.13^e^	3.50 ± 0.09^d^	3.81 ± 0.13^c^	4.23 ± 0.09^b^	4.23 ± 0.09^b^	3.47 ± 0.09^d^	4.26 ± 0.09^b^
k_Rest_ (mg l^–1^ h^–1^)	NA	NA	NA	NA	NA	NA	NA	NA	111 ± 8	114 ± 8	145 ± 6
P_Glc_	NA	NA	NA	NA	NA	NA	NA	NA	2.19 ± 0.08	NA	NA
Glycerol/ethanol (mmol mol^–1^)	45 ± 1^abc^	40 ± 2^d^	45 ± 1^abc^	43 ± 1^bcd^	46 ± 1^ab^	44 ± 1^abc^	44 ± 1^abc^	45 ± 1^abc^	44 ± 1^abc^	47 ± 1^a^	42 ± 1^cd^
Lactic acid/ethanol (mmol mol^–1^)	48 ± 1^bcd^	64 ± 4^a^	44 ± 1^d^	45 ± 1^d^	47 ± 2^bcd^	50 ± 1^bc^	47 ± 1^bcd^	46 ± 1^cd^	46 ± 1^cd^	51 ± 1^b^	44 ± 1^d^
Acetic acid/ethanol (mmol mol^–1^)	31 ± 1^bc^	43 ± 3^a^	33 ± 1^b^	32 ± 1^bc^	31 ± 1^bc^	33 ± 1^bc^	31 ± 1^bc^	30 ± 1^c^	31 ± 1^bc^	32 ± 1^bc^	32 ± 1^bc^
Acetic acid/lactic acid (mmol mol^–1^)	652 ± 19^bc^	673 ± 46^bc^	751 ± 21^a^	715 ± 27^ab^	657 ± 35^bc^	665 ± 19^bc^	653 ± 19^bc^	652 ± 20^bc^	667 ± 22^bc^	626 ± 19^c^	721 ± 25^ab^

*The results are presented as the mean ± standard deviation and significant differences (*p* < 0.05) between different fermentation series are indicated with different superscripts a, b, c, d, e, f, g, h, and i. Abbreviations are as in Table 1. NA, not available.*

The models describing the production of metabolites during water kefir fermentation are illustrated for fermentation series 2S-2G-An and 2S-2L-An (Figure 4). The volumetric production rates of ethanol (k_Eth_), glycerol (k_Gly_), lactic acid (k_LA_), and acetic acid (k_AA_) increased with the concentration of the grain inoculum, but less than expected from the increases in the concentrations of the grain inoculum (Table 3). The volumetric metabolite production rates in the fermentation series 2S-2L-An were around half of those in the fermentation series 2S-2G-An, except for the volumetric production rate of mannitol, which was almost zero in 2S-2L-An. They increased with the concentrations of the water kefir grain inoculum added. The concentrations of sucrose did not substantially impact the production of metabolites. When sucrose was substituted with glucose and fructose in fermentation series 2S-2G-An, 2SGF-2G-An, and 2GF-2G-An, the volumetric production rates of ethanol, glycerol, lactic acid, and acetic acid decreased. Furthermore, the volumetric production rates of ethanol, glycerol, lactic acid, and acetic acid were higher with glucose (2G-2G-An) than with fructose (2F-2G-An), whereas the volumetric production rate of mannitol was higher with fructose (2F-2G-An). The volumetric production rates of ethanol, glycerol, and acetic acid were higher under aerobic fermentation conditions (2S-2G-Ae), whereas those for lactic acid and mannitol were higher under anaerobic fermentation conditions (2S-2G-An). The fermentation series inoculated with a liquor inoculum had the lowest ratios of the volumetric production rates of glycerol to ethanol, and the highest ones of lactic acid to ethanol and acetic acid to ethanol. The highest ratios of the volumetric production rates of acetic acid to lactic acid were found for the aerobic fermentation series.

The fraction of the total metabolism used for the production of metabolites and products that could not be measured was higher with fructose (2F-2G-An) than with glucose (2G-2G-An) or glucose and fructose (2GF-2G-An) (Table 3). When the concentrations of glucose and fructose were similar (2GF-2G-An), glucose was consumed approximately twice as fast as fructose.

### Aroma Compounds

The main higher alcohols found *via* SH-GC-MS were 2-methyl-1-propanol, isoamyl alcohol, and 2-phenylethanol, and the main esters were ethyl acetate, isoamyl acetate, ethyl hexanoate, ethyl octanoate, and ethyl decanoate (Supplementary Figures S4,S5). The production profiles of 2-methyl-1-propanol and isoamyl alcohol followed those of ethanol. In contrast, the concentrations of ethyl acetate, ethyl decanoate, and to a lesser extent ethyl hexanoate increased only slowly during the first 24 to 48 h of all fermentation series, after which their concentrations increased faster. The concentrations of ethyl octanoate increased quickly in all fermentation series and decreased after 72 h of fermentation, whereby the decrease was most pronounced in the aerobic fermentation series. Also, the concentrations of ethyl hexanoate decreased noticeably in the aerobic fermentation series after 72 h of fermentation. The production of ethyl decanoate increased with the time of fermentation, and its concentrations after 96 h of fermentation were higher in the fermentation series 2S-2L-An than in 2S-2G-An. Overall, the production of esters was lower in the fermentation series with fructose than in the fermentation series with glucose or sucrose.

## Discussion

Water kefir fermentation is commonly started with water kefir grains as inoculum (Waldherr et al., 2010; Gulitz et al., 2011, 2013; Laureys and De Vuyst, 2014, 2017; Laureys et al., 2017, 2018, 2019). Yet, several questions about the exact nature and role of these grains during water kefir fermentation remained to be addressed (Laureys et al., 2017, 2018, 2019). The present study contributed to a better characterization of the properties of the water kefir grains by determining their density and microbial colonization, and of their function during water kefir fermentation. The latter was realized by applying a modeling strategy to describe the production of water kefir grain wet mass (expressed as EPS produced) as a function of time during the fermentation process.

The state-of-the-art equipment used in the present study allowed to show that the water kefir microorganisms were predominantly attached onto the surface of the water kefir grains, whereby the yeasts and LAB were not structurally arranged around each other. These results were in line with an early report on the microscopic investigation of tibi grains (Moinas et al., 1980). The main yeasts and LAB species were *S. cerevisiae*, *D. bruxellensis*, *Liql. nagelii, Lacc. paracasei*, and *Lenl*. *hilgardii*, whereby the relative abundances of *S. cerevisiae* and *Lenl*. *hilgardii* were higher on the grains than in the liquors. The main AAB species were *G. roseus*/*oxydans*, *A. fabarum*, and *A. indonesiensis*, which were all found in water kefir before (Gulitz et al., 2011, 2013; Laureys and De Vuyst, 2017). The LAB and yeasts were always the most prevalent microorganisms during the water kefir fermentation processes studied, and they were predominantly associated with the water kefir grains, confirming previous data (Laureys and De Vuyst, 2014, 2017; Laureys et al., 2018, 2019). In contrast, the AAB only proliferated under aerobic fermentation conditions and were always predominantly associated with the water kefir liquors. The proliferation of AAB resulted in high concentrations of acetic acid and ethyl acetate, and low concentrations of higher esters, confirming previous results (Laureys et al., 2018). Similarly, proliferation of AAB in wine results in high concentrations of ethyl acetate and loss of fruity aromas (Bartowsky et al., 2003). High concentrations of acetic acid or ethyl acetate are probably not desired in water kefir, as acetic acid can contribute a harsh acidic taste and aroma, and ethyl acetate a solvent-like aroma. In contrast, higher esters will be desirable in water kefir, as they can contribute fruity aromas (Lambrechts and Pretorius, 2000).

The majority of the metabolic activity of the microorganisms was associated with the grains, and the water kefir fermentation rate increased with the concentration of the water kefir grain inoculum. However, the increase in fermentation rate was less than expected from the increase in the concentration of the water kefir grain inoculum. Indeed, substantial metabolic activity was also found in the water kefir liquor. As an innovative approach, water kefir liquor was used as alternative inoculum to start a water kefir fermentation process, without the production of water kefir grain mass. However, the volumetric production rates for ethanol, glycerol, lactic acid, and acetic acid during a water kefir fermentation process inoculated with liquor were only half of those during a comparable fermentation process inoculated with grains. The production of mannitol was mainly associated with the grains and was negligible when inoculated with liquor. This corresponded with the higher relative abundance of *Lenl*. *hilgardii* on the grains than in the liquors, an obligate heterofermentative LAB species that is able to reduce fructose to mannitol (Verce et al., 2019). Furthermore, starting a water kefir fermentation process with liquor instead of grains resulted in higher viable counts of AAB, as these microorganisms were predominantly associated with the liquor, and this was reflected in high ratios of acetic acid to ethanol.

The water kefir grain growth could be decreased by substituting sucrose (partly) with glucose and/or fructose. Glucose was the preferred alternative substrate, as it was fermented faster than fructose. Indeed, *S*. *cerevisiae* and most LAB ferment glucose more quickly than fructose (Berthels et al., 2004; Endo, 2012), although the growth and metabolism of *Lenl*. *hilgardii* was reported to be faster with fructose than with sucrose or glucose as substrates (Leroi and Pidoux, 1993). Furthermore, when fructose was the substrate during water kefir fermentation, the production of non-measured metabolites and/or other compounds was higher than with sucrose or glucose. Complete substitution of sucrose with glucose and/or fructose resulted in the absence of water kefir grain growth and in lower relative abundances of *Lenl*. *hilgardii* in the water kefir liquors. The latter may be undesirable on the long term, as it might compromise the potential for water kefir grain growth. Low water kefir grain growth decreases the size of the water kefir grains because they are brittle and break easily (Laureys and De Vuyst, 2017; Laureys et al., 2019). This makes them more difficult to sieve and increases their viable counts of microorganisms, resulting in an unstable production process (Laureys et al., 2017).

Sometimes the fast production of water kefir grain wet mass might be desirable. The specific water kefir grain production rate increased with increasing concentrations of the grain inoculum, which was probably caused by a shift of the dextran sucrase activity from sucrose hydrolysis toward dextran biosynthesis at higher dextran concentrations (Mooser et al., 1985). The specific water kefir grain production rate decreased slightly with increasing sucrose concentrations, which was probably caused by substrate inhibition of the dextran sucrases by sucrose concentrations above 36 g l^–1^ (Hehre, 1946). The highest water kefir grain growth was obtained when the concentration of the grain inoculum was lowest, as this minimized acidic stress, substrate inhibition, and substrate depletion (Laureys et al., 2019). The water kefir grain growth may thus be maximized with moderate sucrose concentrations and low concentrations of grain inoculum.

In conclusion, yeasts and LAB were always the most prevalent microorganisms during water kefir fermentation. They were mainly found on the surface of the water kefir grains. In contrast, AAB proliferated only under aerobic fermentation conditions and were mainly found in the water kefir liquors. The water kefir fermentation rate could be increased by increasing the concentration of the grain inoculum, as the majority of the microbial metabolic activity was associated with the water kefir grains. Nevertheless, substantial microbial metabolic activity was also found in the water kefir liquors. Moreover, the water kefir liquor could be used as an alternative inoculum to start a water kefir fermentation process, whereby no water kefir grain wet mass was produced. However, the volumetric production rates of most metabolites (and especially mannitol) were lower when the fermentation processes were inoculated with liquor instead of grains. The production of water kefir grains could be controlled by (partly) substituting sucrose with glucose and/or fructose, whereby glucose was fermented faster than fructose.

## Data Availability Statement

The raw data supporting the conclusions of this article will be made available by the authors, without undue reservation.

## Author Contributions

DL, FL, and LDV designed the study, performed the experiments, acquired the experimental data (except for the culture-dependent microbial species diversity analyses), interpreted the data, performed the statistical analyses, revised, and edited the manuscript. MA and PV performed the culture-dependent microbial species diversity analyses. TH and MR performed the visualizations with the scanning electron microscope. DL and LDV wrote the manuscript in consultation with MA, PV, TH, MR, and FL. All authors approved the final version of the manuscript.

## Conflict of Interest

The authors declare that the research was conducted in the absence of any commercial or financial relationships that could be construed as a potential conflict of interest.

## References

[B1] Bartowsky E. J. Xia D. Gibson R. L. Fleet G. H. Henschke P. A. (2003). Spoilage of bottled red wine by *acetic acid bacteria*. *Lett. Appl. Microbiol.* 36 307–314. 10.1046/j.1472-765X.2003.01314.x 12680944

[B2] Berthels N. J. Cordero Otero R. R. Bauer F. F. Thevelein J. M. Pretorius I. S. (2004). Discrepancy in glucose and fructose utilisation during fermentation by *Saccharomyces cerevisiae* wine yeast strains. *FEMS Yeast Res.* 4 683–689. 10.1016/j.femsyr.2004.02.005 15093771

[B3] Cottet C. Ramirez-Tapias Y. A. Delgado J. F. de la Osa O. Salvay A. G. Peltzer M. A. (2020). Biobased materials from microbial biomass and its derivatives. *Materials* 13:1263. 10.3390/ma13061263 32168751 PMC7143539

[B4] Eckel V. P. L. Vogel R. F. (2020). Genomic and physiological insights into the lifestyle of *Bifidobacterium* species from water kefir. *Arch. Microbiol.* 202 1627–1637. 10.1007/s00203-020-01870-7 32266422

[B5] Eckel V. P. L. Ziegler L. M. Vogel R. F. Ehrmann M. (2020). *Bifidobacterium tibiigranuli* sp. nov. isolated from homemade water kefir. *Int. J. Syst. Evolutionary Microbiol.* 70 1562–1570. 10.1099/ijsem.0.003936 31860428

[B6] Endo A. (2012). Fructophilic lactic acid bacteria inhabit fructose-rich niches in nature. *Microbial. Ecol. Health Dis.* 23:18563. 10.3402/mehd.v23i0.18563 23990834 PMC3747758

[B7] Gulitz A. Stadie J. Ehrmann M. A. Ludwig W. Vogel R. F. (2013). Comparative phylobiomic analysis of the bacterial community of water kefir by 16S rRNA gene amplicon sequencing and ARDRA analysis. *J. Appl. Microbiol.* 114 1082–1091. 23289707 10.1111/jam.12124

[B8] Gulitz A. Stadie J. Wenning M. Ehrmann M. A. Vogel R. F. (2011). The microbial diversity of water kefir. *Int. J. Food Microbiol.* 151 284–288. 10.1016/j.ijfoodmicro.2011.09.016 22000549

[B9] Hehre E. J. (1946). Studies on the enzymatic synthesis of dextran from sucrose. *J. Biol. Chem.* 163 221–233. 21023643

[B10] Horisberger M. (1969). Structure of the dextran of the tibi grain. *Carbohydrate Res.* 10 379–385.

[B11] Hsieh H. H. Wang S. Y. Chen T. L. Huang Y. L. Chen M. J. (2012). Effects of cow’s and goat’s milk as fermentation media on the microbial ecology of sugary kefir grains. *Int. J. Food Microbiol.* 157 73–81. 10.1016/j.ijfoodmicro.2012.04.014 22578996

[B12] Lambrechts M. G. Pretorius I. S. (2000). Yeast and its importance to wine aroma: a review. *S Afr. J. Enol. Viticult.* 21 97–129.

[B13] Laureys D. Aerts M. Vandamme P. De Vuyst L. (2018). Oxygen and diverse nutrients influence the water kefir fermentation process. *Food Microbiol.* 73 351–361. 10.1016/j.fm.2018.02.007 29526223

[B14] Laureys D. Aerts M. Vandamme P. De Vuyst L. (2019). The buffer capacity and calcium concentration of water influence the microbial species diversity, grain growth, and metabolite production during water kefir fermentation. *Front. Microbiol.* 10:2876. 10.3389/fmicb.2019.02876 31921054 PMC6923659

[B15] Laureys D. De Vuyst L. (2014). Microbial species diversity, community dynamics, and metabolite kinetics of water kefir fermentation. *Appl. Environ. Microbiol.* 80 2564–2572. 10.1128/aem.03978-13 24532061 PMC3993195

[B16] Laureys D. De Vuyst L. (2017). The water kefir grain inoculum determines the characteristics of the resulting water kefir fermentation process. *J. Appl. Microbiol.* 122 719–732. 10.1111/jam.13370 27930854

[B17] Laureys D. Cnockaert M. De Vuyst L. Vandamme P. (2016). *Bifidobacterium aquikefiri* sp. nov. isolated from water kefir. *Int. J. Syst. Evolutionary Microbiol.* 66 1281–1286. 10.1099/ijsem.0.000877 26739269

[B18] Laureys D. Van Jean A. Dumont J. De Vuyst L. (2017). Investigation of the instability and low water kefir grain growth during an industrial water kefir fermentation process. *Appl. Microbiol. Biotechnol.* 101 2811–2819. 10.1007/s00253-016-8084-5 28070662

[B19] Leroi F. Pidoux M. (1993). Characterization of interactions between *Lactobacillus hilgardii* and *Saccharomyces florentinus* isolated from sugary kefir grains. *J. Appl. Bacteriol.* 74 54–60. 10.1111/j.1365-2672.1993.tb02996.x 8642011

[B20] Marsh A. J. O’Sullivan O. Hill C. Ross R. P. Cotter P. D. (2013). Sequence-based analysis of the microbial composition of water kefir from multiple sources. *FEMS Microbiol. Lett.* 348 79–85. 10.1111/1574-6968.12248 24004255

[B21] Moinas M. Horisberger M. Bauer H. (1980). The structural organization of the tibi grain as revealed by light, scanning and transmission microscopy. *Arch. Microbiol.* 128 157–161. 10.1007/Bf00406153

[B22] Monsan P. Bozonnet S. Albenne C. Joucla G. Willemot R. M. Remaud-Simeon M. (2001). Homopolysaccharides from lactic acid bacteria. *Int. Dairy J.* 11 675–685. 10.1016/S0958-6946(01)00113-3

[B23] Mooser G. Shur D. Lyou M. Watanabe C. (1985). Kinetic studies on dextransucrase from the cariogenic oral bacterium *Streptococcus mutans*. *J. Biol. Chem.* 260 6907–6915. 2581961

[B24] Neve H. Heller K. J. (2002). The microflora of water kefir: a glance by scanning electron microscopy. *Kieler Milchwirtschaftliche Forschungsberichte* 54 337–349.

[B25] Pidoux M. (1989). The microbial flora of sugary kefir grain (the gingerbeer plant): biosynthesis of the grain from *Lactobacillus hilgardii* producing a polysaccharide gel. *J. Appl. Microbiol.* 5 223–238. 10.1007/Bf01741847

[B26] Pothakos V. Illeghems K. Laureys D. Spitaels F. Vandamme P. De Vuyst L. (2016). “Acetic acid bacteria in fermented food and beverage ecosystems,” in *Acetic Acid Bacteria: Ecology and Physiology*, eds Matsushita K. Toyama H. Tonouchi N. Okamoto-Kainuma A., (Tokyo: Springer), 73–100.

[B27] Romero-Luna H. E. Peredo-Lovillo A. Hernandez-Mendoza A. Hernandez-Sanchez H. Cauich-Sanchez P. I. Ribas-Aparicio R. M. (2020). Probiotic potential of *Lactobacillus paracasei* CT12 isolated from water kefir grains (tibicos). *Curr. Microbiol*. 77 2584–2592. 10.1007/s00284-020-02016-0 32372103

[B28] Verce M. De Vuyst L. Weckx S. (2019). Shotgun metagenomics of a water kefir fermentation ecosystem reveals a novel *Oenococcus* species. *Front. Microbiol.* 10:479. 10.3389/fmicb.2019.00479 30918501 PMC6424877

[B29] Verce M. De Vuyst L. Weckx S. (2020). The metagenome-assembled genome of Candidatus *Oenococcus aquikefiri* from water kefir represents the species *Oenococcus sicerae*. *Food Microbiol.* 88:103402. 10.1016/j.fm.2019.103402 31997765

[B30] Waldherr F. W. Doll V. M. Meissner D. Vogel R. F. (2010). Identification and characterization of a glucan-producing enzyme from *Lactobacillus hilgardii* TMW 1.828 involved in granule formation of water kefir. *Food Microbiol.* 27 672–678. 10.1016/j.fm.2010.03.013 20510787

[B31] Zajšek K. Goršek A. (2010). Modelling of batch kefir fermentation kinetics for ethanol production by mixed natural microflora. *Food Bioprod. Process.* 88 55–60. 10.1016/j.fbp.2009.09.002

