# Supplementary material for: The Type and Concentration of Inoculum and Substrate as Well as the Presence of Oxygen Impact the Water Kefir Fermentation Process
Source: Front Microbiol. 2021 Feb 11;12:628599. doi: 10.3389/fmicb.2021.628599 (PMC7904701; doi:10.3389/fmicb.2021.628599)
Supplement: Supplementary file 1 [file Data_Sheet_1.docx]

Supplementary Materials

# Supplementary figures

**Figure S1.** Water kefir grain wet mass (g), water kefir grain growth (%), and pH as a function of time during water kefir fermentation series 2S-2G-An (●──), 2S-2G-Ae (●- - -), 2S-2L-An (♦──), 2S-1G-An (●──), 2S-3G-An (●──), 1S-2G-An (○──), 3S-2G-An (●──), 2SGF-2G-An (■──), 2GF-2G-An (▲──), 2G-2G-An (▲──), and 2F-2G-An (Δ──). Abbreviations are as in Table 1.

**Figure S2.** Concentrations of sucrose, glucose, and fructose as a function of time during water kefir fermentation series 2S-2G-An (●──), 2S-2G-Ae (●- - -), 2S-2L-An (♦──), 2S-1G-An (●──), 2S-3G-An (●──), 1S-2G-An (○──), 3S-2G-An (●──), 2SGF-2G-An (■──), 2GF-2G-An (▲──), 2G-2G-An (▲──), and 2F-2G-An (Δ──). Abbreviations are as in Table 1.

**Figure S3.** Concentrations of ethanol, glycerol, lactic acid, acetic acid, and mannitol as a function of time during water kefir fermentation series 2S-2G-An (●──), 2S-2G-Ae (●- - -), 2S-2L-An (♦──), 2S-1G-An (●──), 2S-3G-An (●──), 1S-2G-An (○──), 3S-2G-An (●──), 2SGF-2G-An (■──), 2GF-2G-An (▲──), 2G-2G-An (▲──), and 2F-2G-An (Δ──). Abbreviations are as in Table 1.

**Figure S4.** Concentrations of 2-methyl-1-propanol, isoamyl alcohol, 2-phenylethanol, and ethyl acetate as a function of time during water kefir fermentation series 2S-2G-An (●──), 2S-2G-Ae (●- - -), 2S-2L-An (♦──), 2S-1G-An (●──), 2S-3G-An (●──), 1S-2G-An (○──), 3S-2G-An (●──), 2SGF-2G-An (■──), 2GF-2G-An (▲──), 2G-2G-An (▲──), and 2F-2G-An (Δ──). Abbreviations are as in Table 1.

**Figure S5.** Concentrations of isoamyl acetate, ethyl hexanoate, ethyl octanoate, and ethyl decanoate as a function of time during water kefir fermentation series 2S-2G-An (●──), 2S-2G-Ae (●- - -), 2S-2L-An (♦──), 2S-1G-An (●──), 2S-3G-An (●──), 1S-2G-An (○──), 3S-2G-An (●──), 2SGF-2G-An (■──), 2GF-2G-An (▲──), 2G-2G-An (▲──), and 2F-2G-An (Δ──). Abbreviations are as in Table 1.
